# High-speed maskless nanolithography with visible light based on photothermal localization

**DOI:** 10.1038/srep43892

**Published:** 2017-03-02

**Authors:** Jingsong Wei, Kui Zhang, Tao Wei, Yang Wang, Yiqun Wu, Mufei Xiao

**Affiliations:** 1 Laboratory of High-Density Optical Storage, Shanghai Institute of Optics and Fine Mechanics, Chinese Academy of Sciences, Shanghai, 201800, China; 2 University of Chinese Academy of Sciences, Beijing, 100049, China; 3 Centro de Nanociencias y Nanotecnología, Universidad Nacional Autónoma de México, km. 107 Carretera Tijuana-Ensenada, Ensenada, Baja California, CP 22860, México.

## Abstract

High-speed maskless nanolithography is experimentally achieved on AgInSbTe thin films. The lithography was carried out in air at room temperature, with a GaN diode laser (λ = 405 nm), and on a large sample disk of diameter 120 mm. The normal width of the written features measures 46 ± 5 nm, about 1/12 of the diffraction allowed smallest light spot, and the lithography speed reaches 6 ~ 8 m/s, tens of times faster than traditional laser writing methods. The writing resolution is instantaneously tunable by adjusting the laser power. The reason behind the significant breakthrough in terms of writing resolution and speed is found as the concentration of light induced heat. Therefore, the heat spot is far smaller than the light spot, so does the size of the written features. Such a sharp focus of heat occurs only on the selected writing material, and the phenomenon is referred as the photothermal localization response. The physics behind the effect is explained and supported with numerical simulations.

Recently, there has been growing interest in nanostructure-based optical elements that are able to manipulate visible light for various photonic purposes, such as nanosieve^1^, metalens^2^, metamaterial^3^,^4^, and metasurface^5^,^6^,^7^,^8^, etc. Among others, a common feature in these photonic tools is the presence of a large amount of nanostructures (ranging from 200 nm to sub-100 nm in size) distributed usually on a large circular disk (ranging from a few centimeters up to a meter in diameter). Therefore, fabrication of these photonic elements especially for practical industrial applications becomes challenged as conventional lithography techniques can hardly meet the requirements simultaneously.

Nanolithography techniques based on ion/electron beams and soft x-ray^9^,^10^ are widely employed in industrial applications because a shorter wavelength avoids effectively the diffraction limit. However, the need for high-vacuum environment and low speed restricts the techniques to small area patterning. Alternatives, such as stamper-based nanoimprint and mask projection lithography^11^,^12^, are also unrealistic due to the difficulty in fabricating large stampers with nanoscale features. Some scanning probe lithographies^13^,^14^ are able to fabricate nanoscale arbitrary patterns in atmospheric environment, but the lithography remains of low speed and fits only for small area. In view of above, one concludes tentatively that there seems a lack of effective methods for high-speed fabrication of micro/nanostructure-based optical elements on a large disk (see also, for instance, a recent review on maskless lithography in Ref. [15]).

In the present work, we resorted to the widely used laser scanning writing method, where a commercially used GaN diode violet laser at wavelength 405 nm as the light source and an optical lens for focusing the light. On one hand, a direct writing system using a GaN diode laser may solve most of the aforementioned difficulties for high-speed nanolithography as it operates in air, at room temperature, and has virtually no limit on working area and scanning speed; on the other hand, the GaN diode laser is stable and low cost, and the wavelength of 405 nm is close to the limit of visible light. Obviously, the only remaining hurdle is the diffraction limit imposed on the lithography resolution^16^,^17^,^18^, for example, for a writing system operating at 405 nm wavelength with a focusing lens optics of numerical aperture (NA) 0.90, the resolution is limited to about 1.22λ/NA = 550 nm.

In the past years, one also proposed different methods for obtaining below-diffraction-limited pattern structures through using special resists^19^, such as two-photon lithography^20^,^21^, metal hydrazone complex^22^, and two-color irradiation scheme^23^; however, the patterning speed is low and can not meet the real application requirements. The present report shows that the diffraction limit can be overcome by selecting AgInSbTe as the writing material. The reason for the breakthrough stems from a strong photothermal localization response on the surface of the AgInSbTe thin film, so that the heating spot becomes far smaller than the light spot, where the photothermal localization response comes from three aspects, including nonlinear saturation absorption, phase change threshold effect, and the manipulation of thermal diffusion channel. In the rest of the report, we shall first explain the experiment and present experimental results, and then analyze the involved physics with numerical simulations.

## The experiment and experimental results

**Figure 1:**
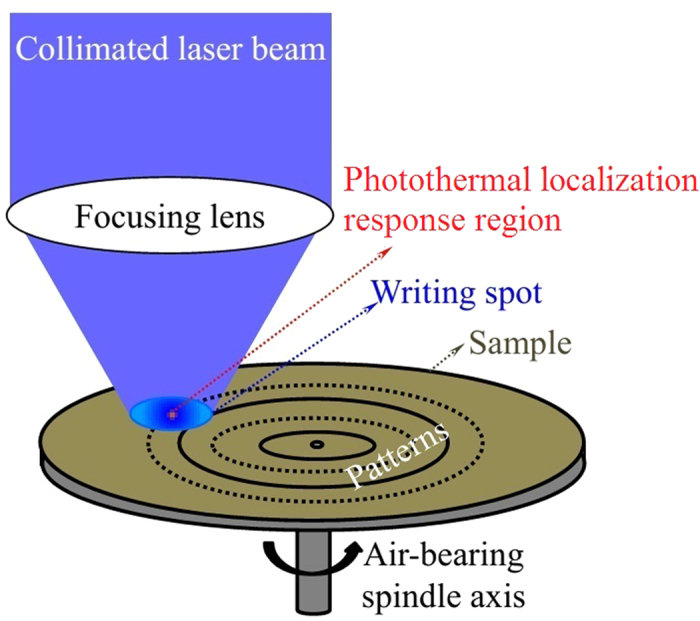
The experiment. Schematic of the high-speed maskless nanolithography on an AgInSbTe coated sample, where heating spot is far smaller than the light spot due to the photothermal localization response.

The nanolithography experiment is illustrated schematically in Fig. 1. The nanolithography is based on a high-speed rotational direct writing system (SpinDWL405)^24^. A collimated beam from a GaN-based diode laser (λ = 405 nm) is focused into an optical writing spot. The writing spot irradiates on the surface of a circular disk of 120 mm in diameter mounted on a rotating stage (the detail of writing system is presented in Supplementary Materials).

The substrate on the disk is glass. An AgInSbTe thin film of 100 nm thick was deposited on the glass substrate through magnetron-controlled sputtering method at room temperature. The sputtering background pressure was approximately 5 × 10^−4^ Pa, the sputtering pressure was about 0.5 Pa in the Ar environment, and the sputtering power was 70 W.

AgInSbTe belongs to Te-based chalcogenide phase change material. In general, the crystal structure is changed and maintained in the AgInSbTe thin film when the laser beam spot irradiates. The lithography pattern is formed on the AgInSbTe thin film with laser marked and unmarked areas. Subsequently, the lithography pattern undergoes a wet-etching process in an ammonium sulfide solution, where the laser-marked area is to be etched off. The pattern and scanning speed can be conveniently controlled by external electronics.

However, we have discovered that when AgInSbTe is used as the writing material, the actual marked spot is considerably smaller than the writing spot, as illuminated in Fig. 1. The phenomenon is referred as the photothermal localization response. Therefore, one expects that the actual writing resolution would break through the diffraction limit. In addition, the writing resolution, thus the size of the heating spot, is instantaneously tunable by tuning the laser power.

**Figure 2:**
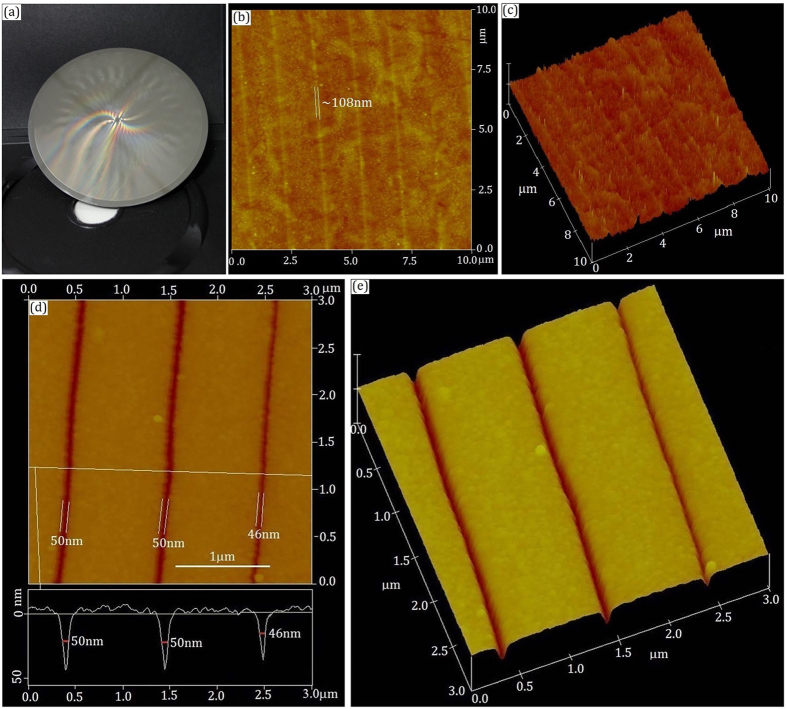
Lithography patterns obtained with a laser power 1.55 mW. (**a**) The morphology of prepared disk with a diameter 120 mm. (**b**) 2D AFM image after laser writing. (**c**) 3D AFM image after laser writing. (**d**) 2D AFM image after wet-etching with a cross-sectional profile. (**e**) 3D AFM image after wet-etching.

Figure 2a is the morphology of prepared disk with a diameter of 120 mm. The laser power was set to P = 1.55 mW, which corresponds to a laser intensity ~1.10 × 10^6^ W/cm^2^. In Fig. 2b and c, 2-dimensional (2D) and 3-dimensional (3D) atomic force microscopy (AFM) images are respectively shown after laser writing. The images are for a small area of the disk, and the written patterns contain a few structural adjustment traces (structure changed area after laser writing). The full linewidth of a trace is measured ~108 nm as indicated in Fig. 2b. The structural adjustment trace presents some slight reliefs as shown in Fig. 2c.

**Figure 3:**
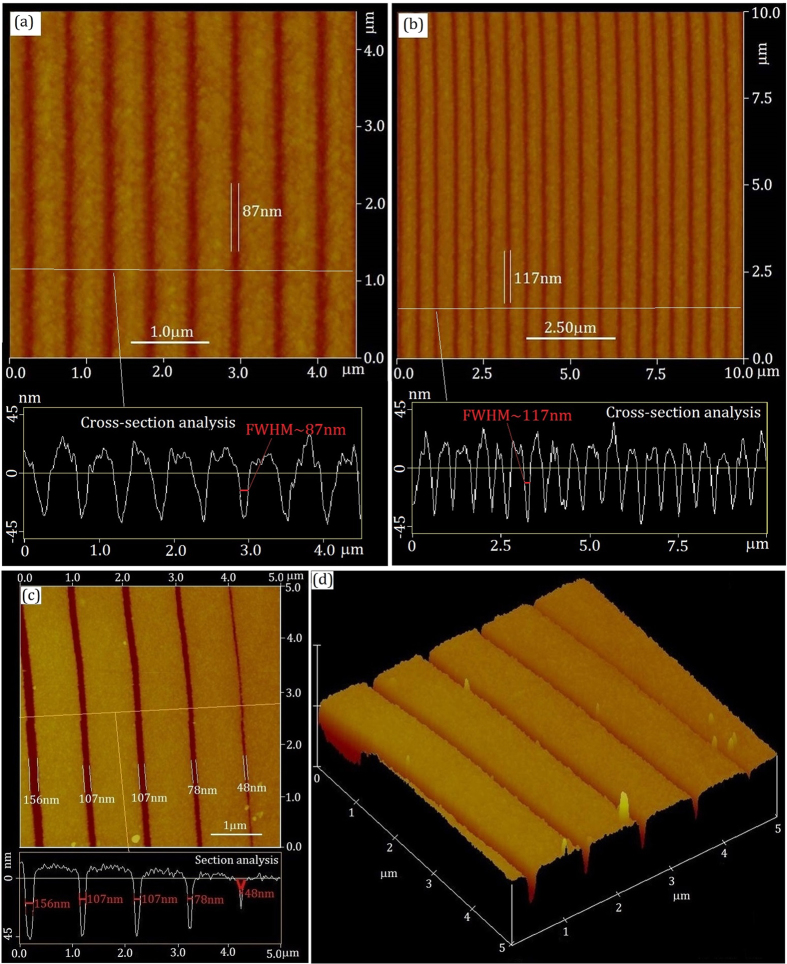
AFM images of Lithography patterns with various laser powers. (**a**) Normal width is 87 ± 8 nm for P = 1.95 mW. (**b**) Normal width is 117 ± 12 nm for P = 2.18 mW. (**c**) Normal width is from 156 nm to 48 nm with rapidly tuned laser power. (**d**) 3D of (**c**).

The written patterns (structural adjustment traces) were further wet-etched by ammonium sulfide solution, the pattern contrast between the written and unwritten areas is enhanced due to the wet-etching selectivity between the laser irradiated and unirradiated areas^25^,^26^,^27^,^28^. In Fig. 2d and f, 2D and 3D AFM images are respectively shown after wet-etching. A cross-sectional profile is inserted in Fig. 2d. One can see that the depth of the line patterns is about 37 nm, and the full width at sample surface is about 100 nm. The minimum full width at half maximum (FWHM) depth, called as normal width, is about 46 ± 5 nm, which is far smaller than the writing spot. The diffraction limited light spot size is about 1.22 λ/NA = 550 nm. The actual spot size was about 600 nm for our laser writing system. Therefore, the achieved minimum size is about 1/12 of the light spot.

An important parameter in our experiment is the laser power. By adjusting the laser power, one is able to tune the pattern resolution instantaneously, as the response time of our system is merely about 10 ns. In Fig. 3, for different laser powers, lithography patterns are presented, where the interval among trench lines is about 0.5 μm. For P = 1.95 mW, the normal width is measured at 87 ± 8 nm as shown in Fig. 3a, whereas for P = 2.18 mW, the normal width increases to 117 ± 12 nm as shown in Fig. 3b. One is readily to conclude that the pattern resolution increases (the size goes down) with the decrease of laser power. However, there is a structural adjustment threshold temperature for AgInSbTe chalcogenides; further improving the pattern resolution by reducing laser power is limited.

In order to demonstrate the capability of our writing system for instantaneously adjusting the pattern resolution, in Fig. 3c and d, 2D and 3D AFM images are presented with five adjacent traces, where the interval among trench lines is about 1.0 μm. Each of the trace was written under a different laser power. From left to right, the laser power is gradually reduced for five traces and the normal width changes accordingly from 156 nm to 48 nm. This was realized when the writing speed was 6 ~ 8 m/s (corresponds to about 48 mm^2^/min. @ 50 nm), which indicates that it is possible to have different pattern resolution for each annular groove. This extraordinary capability should be able to find its photonic applications. In addition, some other lithography results are listed in Supplementary Materials. Based on Fig. 3 and Fig. s3 in Supplementary Materials, one can see that the interval of among trench lines can be arbitrarily tuned through controlling the movement stage.

## The physical mechanisms and numerical simulations

Indeed, the phase change in the writing material is a material reaction to the photothermal effects. When the laser irradiates onto the AgInSbTe thin film, apart from Joule dissipation, the absorbed photons excite electrons and holes. When a part of the photoexcited electrons and holes recombine non-radiatively, the photon energy is transferred into heat, which enables the phase change in the material. In order to have a strong photothermal localization response, i.e. the heating spot far smaller than the light spot, the writing material is desired to possess the following properties: (1) large linear and nonlinear absorption coefficients at the laser wavelength, (2) suitable structural adjustment temperature, (3) etching selectivity between written and unwritten areas.

In the present work, AgInSbTe, a Te-based chalcogenide, is selected as the writing material. We previously measured the nonlinear absorption characteristics of AgInSbTe thin films with an open-aperture mode z-scan method^29^,^30^, and found that the nonlinear absorption coefficient β∼−4.2 × 10^−3^ m/W at 405 nm light wavelength. The fact that β < 0 implies that the AgInSbTe thin film possesses nonlinear saturation absorption (NSA) characteristics. This β value is several orders of magnitude larger than that of common optical materials, which reportedly stems from thermally-induced weakening of resonant bonds in chalcogenides^31^,^32^. In general, the thin films of strong NSA exhibit large linear absorption as well, and the linear absorption coefficient of AgInSbTe is α_0_ ~ 7.63 × 10^7^/m at 405 nm light wavelength.

**Figure 4:**
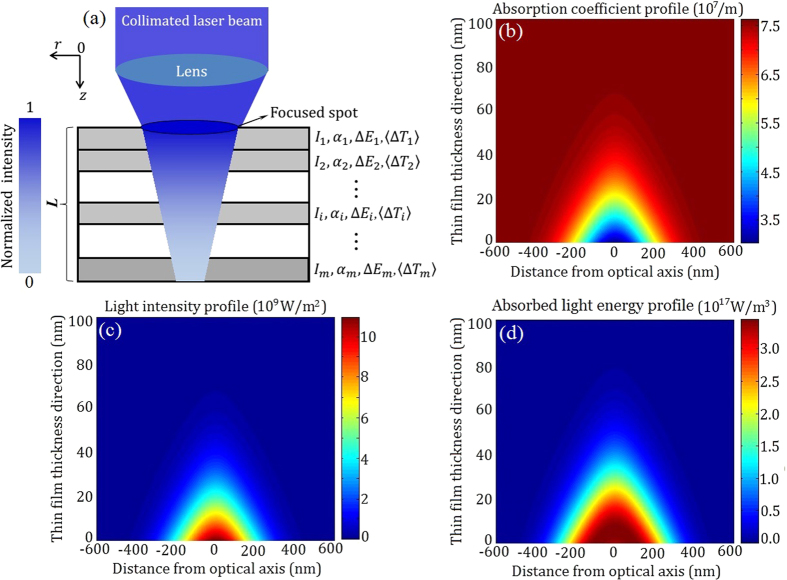
Numerical simulations. (**a**) Schematic of the multilayer model. (**b**) Distribution of absorption coefficient. (**c**) Distribution of light intensity. (**d**) Distribution of absorbed light energy.

With the absorption coefficients and a laser power of P = 1.55 mW, we carried out numerical simulations to determine the steady spatial distributions of light intensity, absorption coefficient, and absorbed light energy, and the results are presented in Fig. 4. The calculation processes are given in Supplementary Materials. When the writing spot with a Gaussian intensity profile irradiates on the sample surface, the light intensity decays exponentially into the thin film as 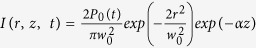, where *z* and *r* are vertical and radial coordinates, respectively, *w*_0_ is the radius of the writing spot at 1/e^2^ maximum intensity, P_0_(*t*) is the instantaneous laser power absorbed by the AgInSbTe thin film surface, and *t* is time. *I* is the laser intensity inside the sample and *α* is the absorption coefficient. For NSA thin films, the absorption coefficient can be written as 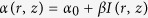 with *β* < 0. The integration over time is realized with a multilayer model as illuminated in Fig. 4a, where the thin film is assumed to be composed of m thin layers of thickness Δ*L* 33,34. When the distributions of light intensity and absorption coefficient are calculated, the absorbed light energy can be estimated by 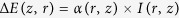.

From the distributions in Fig. 4, one realizes that there is a considerable localization of light and energy at the light spot center. It is known that for chalcogenide phase change materials, the difference of absorption coefficient among different structural states may be of several times^35^.

**Figure 5:**
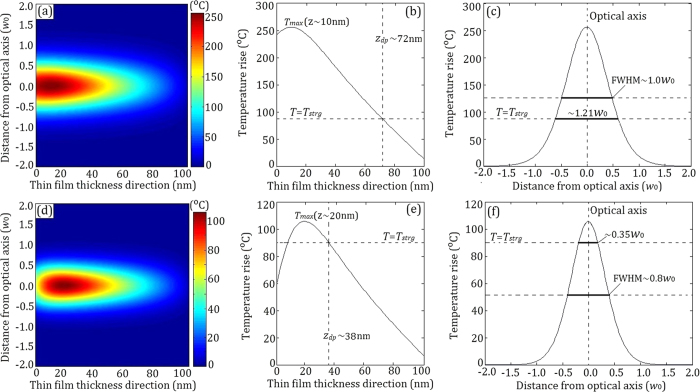
Numerical simulations of photothermal localization response. (**a**) Temperature rise profile of *T (r, z*) with 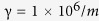. (**b**) Temperature rise profile of 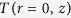 with 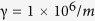. (**c**) Temperature rise profile of 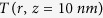 with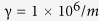. (**d**) Temperature rise profile of 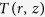 with 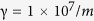. (**e**) Temperature rise profile of 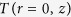 with 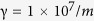. (**f**), Temperature rise profile of 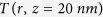 with 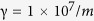.

With the calculated energy profiles in Fig. 4, one is able to further calculate the local temperature rise. The temperature rise profile *T*(*r,z,t*) is determined by solving the coupled transport equations^36^, 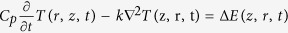 and 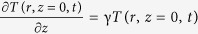 with proper boundary and initial conditions. The sample rotation speed is fixed at *ν* ∼ 8 m/s, and the laser irradiation time at every position is calculated as 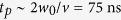. The thermal conductivity 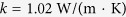 and the heat capacity 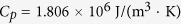
37. The parameter γ is the heat exchange coefficient between the sample surface and the ambient. The 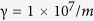 is chosen due to high-speed rotation writing^36^, for comparison, a low-speed rotation writing is also simulated by set a smaller 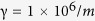, the simulated results are presented in Fig. 5.

Figure 5a–c are for the low-speed case, whereas Fig. 5d–f for the high-speed case (the actual experimental condition). One sees that there appears a significant concentration of heat, as the FWHM is 1.0 *w*_0_ for the low-speed case and 0.8 *w*_0_ for the high-speed case, both are significantly narrower than the FWHM of light spot of 2 *w*_0_. On the other hand, the temperature in the case of low-speed is much higher than that in high-speed case, because the heat is blown away much faster when the laser writing is at a high-speed.

There is a phase change threshold for AgInSbTe thin films. Only when the AgInSbTe thin film is heated beyond the structural relaxation (or glass transition) temperature *T* > *T*_*strg*_ (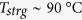)^38^, the structural adjustment takes place. The spot width at the threshold point is marked as 0.35 *w*_0_ (∼105 nm), as shown in Fig. 5f. At the same time, the 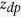 (at *T* > *T*_*strg*_) is extended to about 38 nm, which is larger than the light penetration depth of ∼10 nm. Note that both the width and depth of simulated spot are in consistence with the experimental data in Fig. 2.

Generally speaking, the temperature is the highest at surface and then decreases along the sample thickness direction. However, one can notice that the highest temperature occurs at about *z* ∼ 10 nm in Fig. 5a and b, which originates from the NSA characteristics. The NSA characteristics cause the maximum of absorbed light energy occurred at z  ∼ 10 nm, as shown in Fig. 4d. With the increase of sample movement speed, the heat is blown away much faster from the sample surface, the highest temperature position moves toward the interior of sample, as shown in Fig. 5d and e, where the highest temperature moves to *z* ∼ 20 nm due to thermal diffusion effect. In addition, although the light penetration depth is about 10 nm, the *z*_*dp*_ is extended to about 38 nm, which is due to the thermal diffusion along out-of-plane. The *z*_*dp*_ (∼38 nm) is also basically consistent with the groove depth of about 40 nm, as shown in Fig. 2.

**Figure 6:**
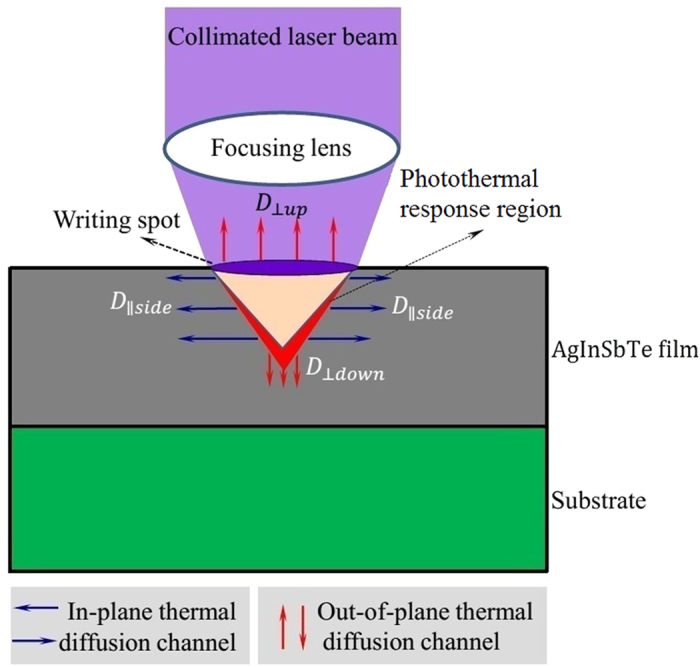
Schematic of thermal diffusion channels.

Actually, there is thermal diffusion influencing the pattern resolution for the high-speed laser writing. The heat diffusion is a little more complicated, because the diffusion is different for different directions (channels). The schematic of thermal diffusion is presented in Fig. 6. When local temperature in the heating spot arises higher than surroundings, heat quickly diffuses into the surroundings. Compared with other sulphur-based chalcogenides or organic resists, AgInSbTe is one of semimetallic semiconductors and possesses a large thermal diffusion coefficient of 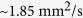
37. The thermal diffusion may cause the pattern resolution to be inferior to the theoretical results. The heat can diffuse along both the in-plane channel (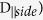 and out-of-plane channel (D_⊥_). The D_⊥_ includes 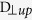 and 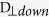, as shown in Fig. 6. The process of photothermal localization response involves both 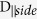 and D_⊥_ channels. The 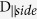 channel is along radial direction, and enlarges the photothermal region, while the D_⊥_ channel diminishes the photothermal region. Therefore, there is a need to attenuate the 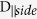 channel and meantime enhance the D_⊥_ channel, which can be realized through a high-speed rotation movement, as a strong air flow along the sample surface is created. The strong air flow makes heat diffusion along out-of-plane easier, and improves the D_⊥*up*_ channel. The D_⊥*up*_ channel removes quickly the excess heat and prevents heat from accumulating in the in-plane region. Especially when the sample rotates very fast, convection losses from the sample surface might become very important. Thereby, the ill-influence of 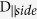 on the pattern resolution can be largely diminished.

In addition, the D_⊥_ channel, including D_⊥*up*_ and D_⊥*down*_, can improve the lithography pattern depth. For AgInSbTe thin films, the light penetration depth is only about 10 nm, which is a very low value for lithography. Without the thermal diffusion, the lithography depth would be restricted to only about 10 nm, because there is no light at a deeper place. Unlike sulphur/selenium-based chalcogenides and organic photoresists, AgInSbTe bears a relatively large thermal diffusion coefficient, which enables the photothermal response to be significantly deeper than the light penetration, and consequently extends the lithography depth.

## Conclusions

In summary, we have proposed and experimentally realized high-speed maskless nanolithography with visible light. The lithography operates in air, at room temperature, and is based on a GaN diode laser. The pattern resolution breaks through the diffraction limit in such a way that the minimum size of the written features is about 1/12 of the size of the diffraction allowed light spot, and the pattern resolution is instantaneously tunable by tuning the laser power. The writing speed reaches 6 ~ 8 m/s, tens of times faster than traditional laser writing methods, and therefore the lithography is suitable for a large sample. The reason for the breakthrough is that we select AgInSbTe as the writing material, and strong photothermal localization response takes place so that the heating spot is far smaller than the light spot. The physical mechanism behind the photothermal localization response is explained and supported by numerical simulations. The simulation results are in consistence with the experimental results. Based on the presented physical mechanism, the pattern feature size may be further reduced through selecting the resist materials with low thermal diffusion coefficient, clear structural change threshold, and strong nonlinear absorption effect.

## Additional Information

**How to cite this article:** Wei, J. *et al*. High-speed maskless nanolithography with visible light based on photothermal localization. *Sci. Rep.* **7**, 43892; doi: 10.1038/srep43892 (2017).

**Publisher's note:** Springer Nature remains neutral with regard to jurisdictional claims in published maps and institutional affiliations.

## Supplementary Material

Supplementary Materials
