# Supplementary material for: High-speed maskless nanolithography with visible light based on photothermal localization
Source: Sci Rep. 2017 Mar 2;7:43892. doi: 10.1038/srep43892 (PMC5333155; doi:10.1038/srep43892)
Supplement: Supplementary Materials [file srep43892-s1.pdf]

Supplementary materials to:

High-speed maskless nanolithography with visible light based on photothermal localization

Jingsong Wei[1*], Kui Zhang[1], Tao Wei[1,2], Yang Wang[1], Yiqun Wu[1], and Mufei Xiao[3]

[1]Laboratory of High-Density Optical Storage, Shanghai Institute of Optics and Fine Mechanics, Chinese Academy of Sciences, Shanghai 201800, China

[2]University of Chinese Academy of Sciences, Beijing 100049, China

[3]Centro de Nanociencias y Nanotecnología, Universidad Nacional Autónoma de México, km. 107 Carretera Tijuana-Ensenada, Ensenada, Baja California CP 22860, México.

[*]Corresponding E-mail: weijingsong@siom.ac.cn

The calculation of photothermal localizaiton effect

The writing material of thickness L is considered as a multi-thin-layer stack (e.g., m layers), and the thickness of every layer is ΔL, $m * \Delta L = L$ (also read Fig. 4a of manuscript). The light intensity and absorption coefficient of the ith layer are marked as $I_i(r,z)$ and $\alpha_i(r,z)$, respectively, where $i = 1, 2, 3, \dots, m$. The absorbed light energy at the ith layer is marked as $\Delta E_i(r,z)$. $\Delta E_i(r,z) = \alpha_i(r,z) \times I_i(r,z)$, where the interference between the stack layers may be neglected due to the strong linear absorption within every layer. The calculation processes are so follows.

The light is focused on the surface of writing material and the intensity profile is written as

$$I_{in}(r,z) = I_0\, exp[-2r^2/w^2(z)],\ w(z) = w_0\sqrt{1 + (z/z_0\ )^2} \quad (1)$$

where $z_0 = \pi w_0^2/\lambda$ is the Rayleigh length. On the writing material surface, $z = 0$, $w(z = 0) = w_0$, and $I_{in} = I_{inc} = I_0 \exp[-2r^2/w_0^2]$. w_0 is the beam waist radius, which can be calculated as $w_0 \sim 0.61\lambda/NA$. The light passes through the 1st layer and decays into the 2nd layer due to the absorption effect. If m is large enough, the exiting intensity for every layer can be calculated using the Beer-Lambert formula.

The 1st layer can be considered as a sample surface, for which the absorption coefficient $\alpha_1(r,z)$ and exiting light intensity $I_1(r,z)$ can be obtained as,

$$\alpha_1(r,z) = \alpha_0 + \beta I_{inc}(r,z), I_1(r,z) = I_{inc}(r,z)e^{-\alpha_1(r,z)\Delta L},$$

$$\Delta E_1(r,z) = \alpha_1(r,z) \times I_1(r,z) \quad (2)$$

where the α_0 and β are linear and nonlinear absorption coefficients of writing material, respectively.

At the ith layer,

$$\alpha_i(r,z) = \alpha_0 + \beta I_{i-1}(r,z), I_i(r,z) = I_{i-1}(r,z)e^{-\alpha_i(r,z)\Delta L},$$

$$\Delta E_i(r,z) = \alpha_i(r,z) \times I_i(r,z) \quad (3)$$

At the last layer, that is, the mth layer,

$$\alpha_m(r,z) = \alpha_0 + \beta I_{m-1}(r,z), I_m(r,z) = I_{m-1}(r,z)e^{-\alpha_m(r,z)\Delta L},$$

$$\Delta E_m(r,z) = \alpha_m(r,z) \times I_m(r,z) \quad (4)$$

The absorbed energy profile inside the writing material can be calculated by the formulas (1)-(4).

The absorbed laser energy (photons) excites the electrons and holes in the writing material.

The photoexcited electrons and holes usually recombine non-radiatively, and the absorbed photon energy is transferred into temperature rise above ambient due to strong electron-phonon coupling. According to formulas (1)-(4), the absorbed laser power unit volume can be rewritten as $\Delta E(r,z,t) = \alpha(r,z)I(r,z,t)$.

Assuming that the heat loss from the sample surface is proportional to temperature rise of $T(r,z=0,t)$, the temperature rise profile $T(r,z,t)$ is determined by the non-steady state heat-conduction eqauation[1]:

$$C_p \frac{\partial}{\partial t} T(r,z,t) - k\nabla^2 T(\mathrm{z},\mathrm{r},\mathrm{t}) = \Delta E(z,r,t) \qquad (5)$$

$$\frac{\partial T(r,z=0,t)}{\partial z} = \gamma T(r,z=0,t) \qquad (6)$$

with the following boundary and initial conditions: $T(r,z,t=0)=0$, $T(r=\infty,z,t) = T(r,z=\infty,t)=0$. The $T(r,z,t)$ and $\partial T(r,z,t)/\partial z$ must be continuous at the boundaries among air, writing material, and substrate. γ is the heat exchange coefficient between sample surface and ambient, and C_p is heat capacity and k is thermal conductivity. $\nabla^2 = \frac{\partial^2}{\partial r^2} + \frac{1}{r}\frac{\partial}{\partial r} + \frac{\partial^2}{\partial z^2}$ is Laplacian operator for circularly symmetric functions. One can numerically resolve the formulas (5) and (6), and the temperature rise profile inside the writing material can be obtained, accordingly.

Establishment of high-speed rotation-type diode-based laser writing system

The basic layout of the constructed direct laser writing system is as follows. The GaN semiconductor diode laser emits a light beam of wavelength 405 nm, the light beam passes through a beam expander and is reflected onto a lens with a numerical aperture (NA) of 0.90.

The light beam is focused on the sample surface by the lens, and the diameter of focused spot is theoretically 550 nm. The real spot diameter is about 600 nm since the optical system is not perfect. The sample is placed on a vacuum absorption stage, which has the diameter of 120 mm. The vacuum sorption stage can be driven by an air-bearing rotation motor at high speed with maximum linear speed of 10 m/s, and the rotation motor is controlled by a computer. The rotation movement angle (θ) is quickly measured by a circular grating ruler and then fed to the computer. The radial movement is provided by a linear motor, which is also controlled by the computer. The linear movement distance (marked as *r*) is measured by a linear grating ruler and then fed to the computer. Therefore, the laser beam spot position on the sample can be rapidly obtained as a polar coordinate (θ, *r*).

The laser spot is easy to defocus in the high-speed writing process due to the influence of sample surface flatness, external vibration, the fluctuation of movement stage, *etc*. To overcome these obstacles, the autotracking module based on astigmatic method is installed in rotation-type direct laser writing system[2]. The autotracking module can find the focus displacement of the sample by detecting the focus error signal and make the sample always be located in the focal plane. Figure s1 is the real picture of established system in this work.

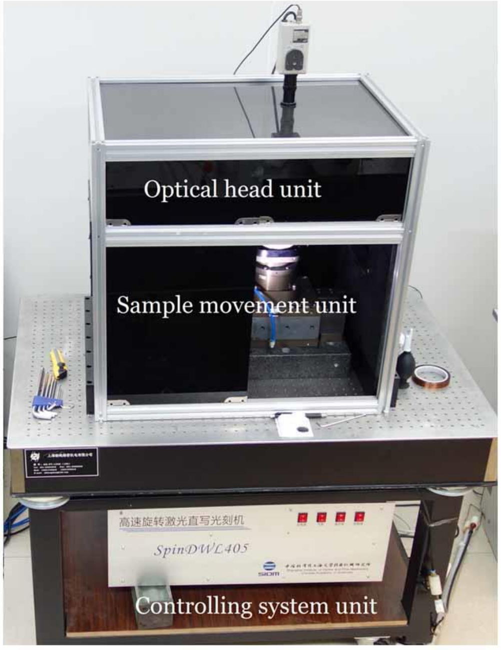

Figure s1 | Real image of high-speed rotation-type diode-based laser writing system.

Some other results on high-speed maskless lithpgraphy

In the process of photothermal localization lithography, the line patterns with different linewidth were directly obtained through rapidly changing the laser power. Figure s2 gives the experimental results. In order to enhance the pattern contrast between the written and original areas, the written patterns were further wet-etched by ammonium sulfide solution. Figure s2(a) presents the two-dimensional AFM imaging and cross-section analysis, and the inset is the height of the line patterns. The three-dimensional AFM image is presented in Figure s2(b). One can see that the line patterns are very clear, and the different linewidth patterns are written on the AgInSbTe thin films, and the FWHM depth changes from 250 nm to 50 nm through quickly changing the laser intensity, accordingly.

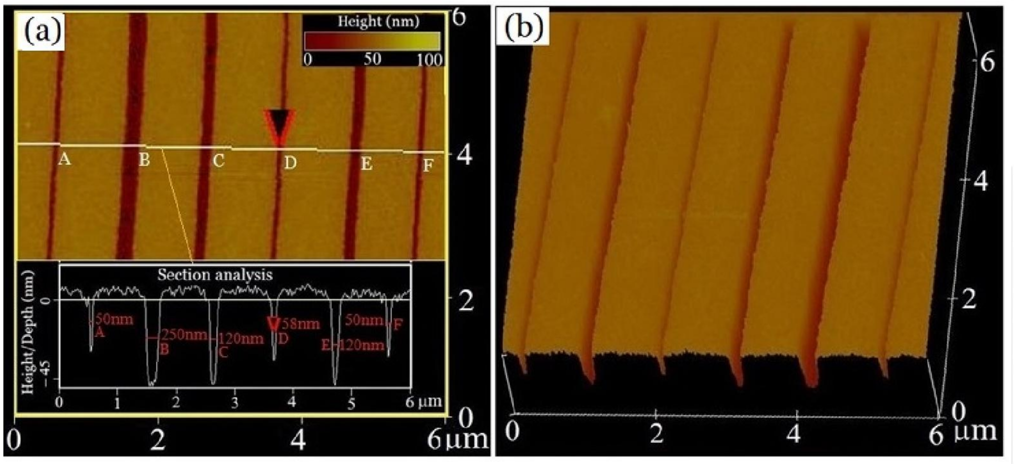

Figure s2 | Experimental results of line patterns with different linewidth through rapidly changing the laser power. (**a**) Two-dimensional AFM images, and (**b**) three-dimensional AFM images.

Large area nanoscale line patterns are also obtained in our experiments, Figures s3a and s3b are SEM images of lithography lines with FWHM depth (70 ± 7) nm and (98 ± 10) nm, respectively.

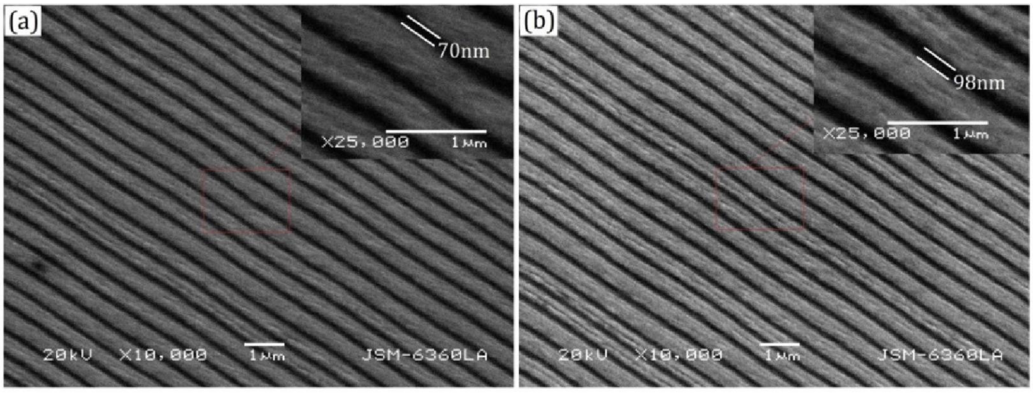

Figure s3 | SEM images of lithography lines with FWHM depth (a) (70 ± 7) nm and (b) (98 ± 10) nm**.** The insets are the magnification of lithographic lines.

Generally speaking, it is difficult to fabricate complex (arbitrary) patterns for high-speed rotation polar coordinate laser writing system. However, in our system, one can transfer the patterns into bitmap format. Then the laser writing system automatically transfers bitmap to

polar coordinates. Therefore the complex (arbitrary) patterns can be written in the format of bitmap. Figure s4 presents all kinds of complex patterns obtained through using the high-speed rotation-type laser writing system, where the complex patterns were etched by ammonium sulfide solution. Figure s4a is the lion image, one can see that the appearance and expression of the lion have been fully written on the AgInSbTe thin films. Figure s4b is the gear pattern, and Fig. s4c- s4f are all kinds of snowflake patterns with fine structures.

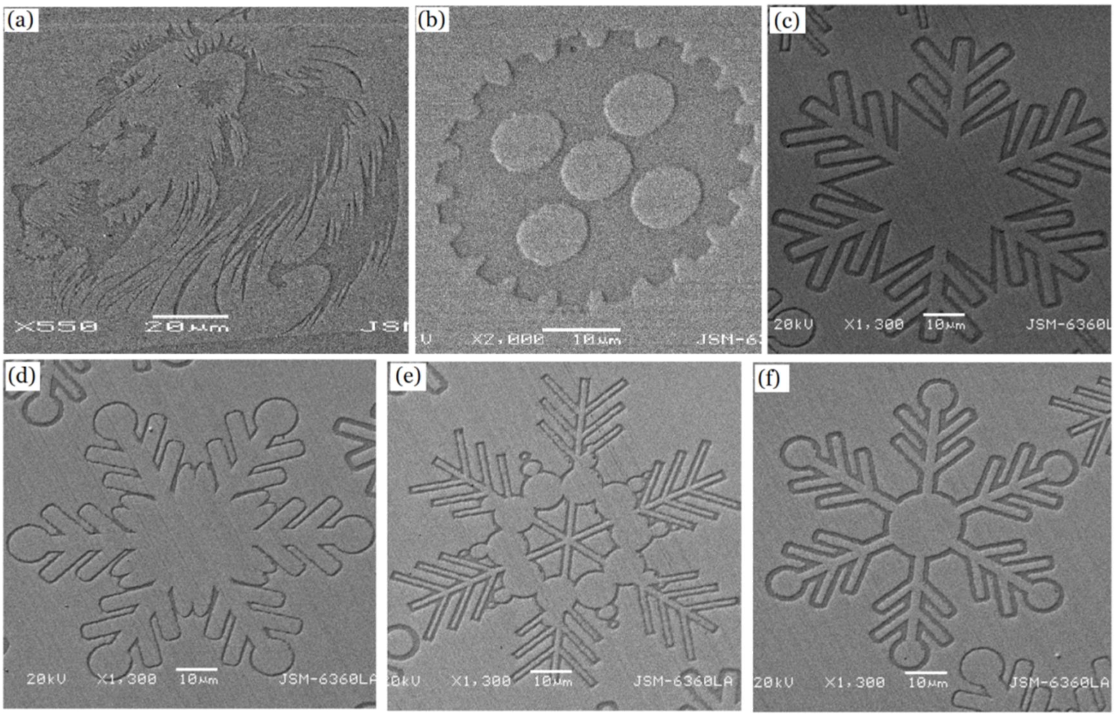

Figure s4 | Complex patterns on AgInSbTe thin films. SEM images (**a**) lion pattern. (**b**) gear patterns, (**c**)-(**f**) different snowflake patterns.

References

1. Mansuripur, M., Neville Connell, G. A. & Goodman, J. W. Laser-induced local heating of multilayers. *Applied Optics* **21**, 1106-1114 (1982).
2. Wei-Yao, H., *et al.* Development of the fast astigmatic auto-focus microscope system. *Measurement Science and Technology* **20**, 045902 (2009).
